# Engineering bacteria to solve the Burnt Pancake Problem

**DOI:** 10.1186/1754-1611-2-8

**Published:** 2008-05-20

**Authors:** Karmella A Haynes, Marian L Broderick, Adam D Brown, Trevor L Butner, James O Dickson, W Lance Harden, Lane H Heard, Eric L Jessen, Kelly J Malloy, Brad J Ogden, Sabriya Rosemond, Samantha Simpson, Erin Zwack, A Malcolm Campbell, Todd T Eckdahl, Laurie J Heyer, Jeffrey L Poet

**Affiliations:** 1 Davidson College, Department of Biology, Davidson, NC 28036, USA; 2 Davidson College, Department of Mathematics, Davidson, NC 28036, USA; 3 Missouri Western State University, Department of Biology, St. Joseph, MO 64507, USA; 4 Missouri Western State University, Department of Computer Science, Math and Physics, St. Joseph, MO 64507, USA; 5 Hampton University, Biology Department, Hampton, VA 23668, USA; 6 Central High School, St. Joseph, MO 64506, USA

## Abstract

**Background:**

We investigated the possibility of executing DNA-based computation in living cells by engineering *Escherichia coli *to address a classic mathematical puzzle called the Burnt Pancake Problem (BPP). The BPP is solved by sorting a stack of distinct objects (pancakes) into proper order and orientation using the minimum number of manipulations. Each manipulation reverses the order and orientation of one or more adjacent objects in the stack. We have designed a system that uses site-specific DNA recombination to mediate inversions of genetic elements that represent pancakes within plasmid DNA.

**Results:**

Inversions (or "flips") of the DNA fragment pancakes are driven by the *Salmonella typhimurium *Hin/*hix *DNA recombinase system that we reconstituted as a collection of modular genetic elements for use in *E. coli*. Our system sorts DNA segments by inversions to produce different permutations of a promoter and a tetracycline resistance coding region; *E. coli *cells become antibiotic resistant when the segments are properly sorted. Hin recombinase can mediate all possible inversion operations on adjacent flippable DNA fragments. Mathematical modeling predicts that the system reaches equilibrium after very few flips, where equal numbers of permutations are randomly sorted and unsorted. Semiquantitative PCR analysis of *in vivo *flipping suggests that inversion products accumulate on a time scale of hours or days rather than minutes.

**Conclusion:**

The Hin/*hix *system is a proof-of-concept demonstration of *in vivo *computation with the potential to be scaled up to accommodate larger and more challenging problems. Hin/*hix *may provide a flexible new tool for manipulating transgenic DNA *in vivo*.

## Background

**Figure 1:**
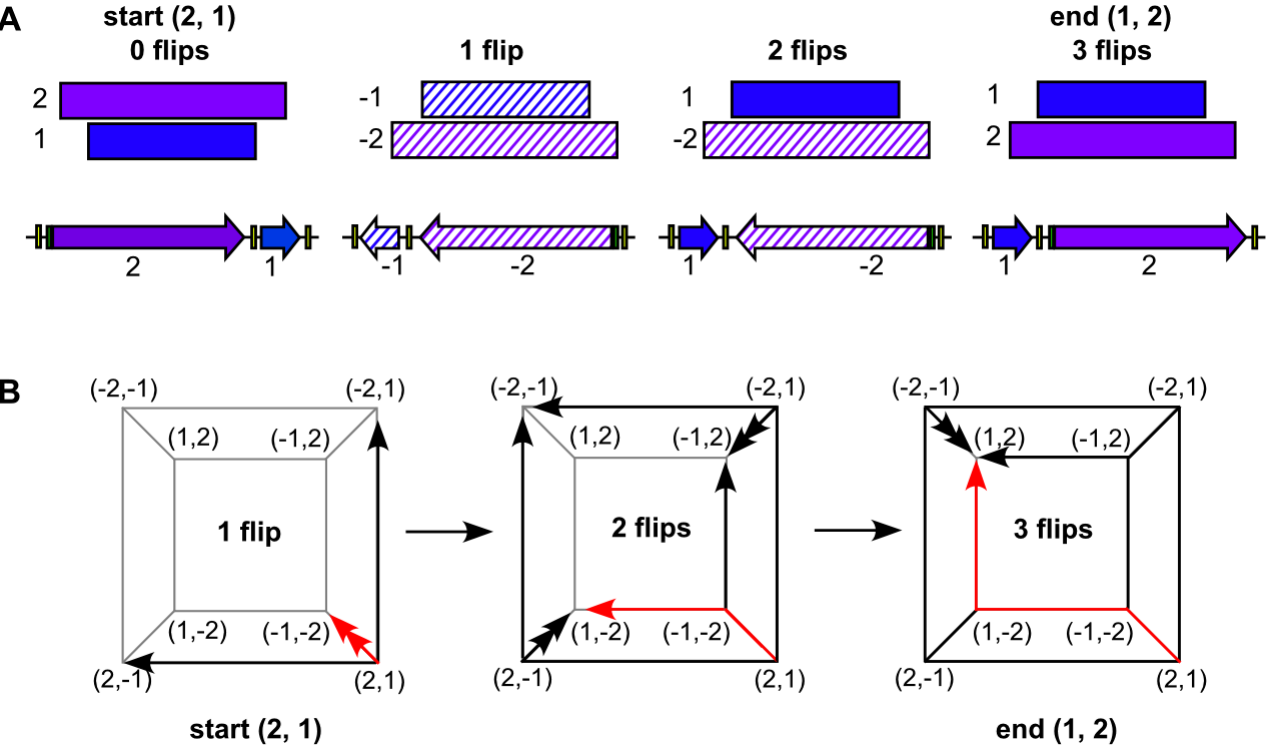
**The Burnt Pancake Problem can be modeled using genetic elements**. (A) Sorting of a scrambled two-pancake stack (rectangles) where the smaller burnt pancake (1, blue) and the larger burnt pancake (2, purple) are in the wrong order (2, 1). First, the whole stack is flipped and both pancakes are turned burnt side up (hatched shading). The next two flips turn the small then the large pancake golden side up (solid shading) resulting in a properly sorted stack (1, 2). Analogous DNA segment arrangements are shown below. Sorting of the promoter (1, blue arrow) and coding region (2, purple arrow) into (1, 2) is required for gene expression. (B) The process of sorting scrambled pancake stack (2, 1) into the solution (1, 2) is plotted on a graph. The eight possible arrangements of the pancake stack are shown as signed permutations at the vertices. (2, 1) is converted into the three neighboring permutations by a flip of a single pancake (arrow) or both pancakes simultaneously (double headed arrow). Six distinct paths of length 3 can convert (2, 1) into (1, 2). The flipping pathway highlighted in red corresponds to the flips shown in part A

The tremendous information storage capacity of DNA and the remarkable efficiency of biomolecular self-assembly have inspired researchers to design biological computers. Previous work has created proof-of-concept biological computers based on *in vitro *self-assembly of DNA [1] and protein-DNA interactions [2-4]. Thus far, biological computing is limited to nonliving devices that have not utilized the parallel processing power afforded by DNA replication and cellular division. In order to demonstrate the feasibility of *in vivo *computing, we programmed *Escherichia coli *to address a classic mathematical challenge called the Burnt Pancake Problem (BPP) [5]. The BPP can be visualized as a stack of different sized pancakes, each having one burnt side and one golden side, arranged in an arbitrary order. The stack must be sorted by flipping individual pancakes or subsets of adjacent pancakes until the pancakes are ordered from smallest to largest with each pancake oriented golden side up (see example in Fig. 1). The BPP is also known as sorting by reversals since both the order and orientation of the pancakes are changed when they are flipped. The BPP is a subject of interest in basic mathematical and computational research (*e.g.*, [5]). Of particular interest to biologists is the application of the BPP to comparative genomics. The evolutionary distance between syntenic genomes of two organisms is determined by the minimum number of reversals required to sort regions of genes in one organism to match the order and orientation of orthologous genes in the other organism [6-8]. The total number of possible arrangements of *n *objects (*i.e.*, pancakes or genes) is 2^*n*^(*n*!), an exponential increase in arrangements as the stack of objects (pancakes or genes) becomes larger. Plasmid DNA replication and exponential cell growth in bacteria are inexpensive, occupy much less space than computer hardware, and maintain parity with the exponential increase in BPP arrangements. Therefore, solving the BPP in living cells offers unique advantages over using computer hardware.

The biological equivalent of a burnt pancake is a functional module of DNA such as a promoter or coding region (Fig. 1a). Similar to burnt pancakes in the BPP, DNA modules have directionality (5' to 3'), require a specific order of the units (*e.g.*, promoter followed by coding region) and can be flipped (cut, inverted, and spliced *in vivo *by cellular machinery). We designed a modular system in which pancake stacks are assembled from flippable DNA segments. Flipping of the DNA segment "pancakes" is mediated by a *Salmonella typhimurium*-derived DNA recombination system. In Salmonella, Hin DNA recombinase catalyzes an inversion reaction that regulates the expression of alternative flagellin genes by switching the orientation of a promoter located on a 1 kb invertible DNA segment [9,10]. Two palindromic 26 bp *hix *sequences flank the invertible DNA segment and serve as the recognition sites for cleavage and strand exchange. A ~70 bp cis-acting *recombinational enhancer *(*RE*) increases efficiency of protein-DNA complex formation [11]. We have reconstituted the genetic elements required for DNA inversion as a collection of modular genetic elements for use in *E. coli*. Our system is a proof-of-concept genetic computing device that manipulates plasmid DNA processors within living cells.

## Results and discussion

### Design and construction of a Hin/*hix*-based DNA recombination system

DNA inversion occurs very rapidly *in vitro*. Protein-DNA complex assembly, strand cleavage, inversion, and ligation occur in less than 1 minute [11]. Therefore, we engineered Hin/*hix *inversion to be more tractable to regulation and kinetic studies by decreasing inversion efficiency. Hin was cloned from *S. typhimurium *by PCR. An *ssrA *LVA protein degradation tag [12] was added to the C-terminal DNA binding domain to prevent over accumulation of Hin and to achieve tighter control of DNA inversion. In Salmonella, the asymmetrical palindromic sequences *hixL *and *hixR *flank the invertible DNA segment and serve as the recognition sites for cleavage and strand exchange. Our system uses *hixC*, a composite symmetrical *hix *site that shows higher binding affinity for Hin and a 16-fold slower inversion rate than wild type sites *hixL *and *hixR *[13,14].

**Figure 2:**
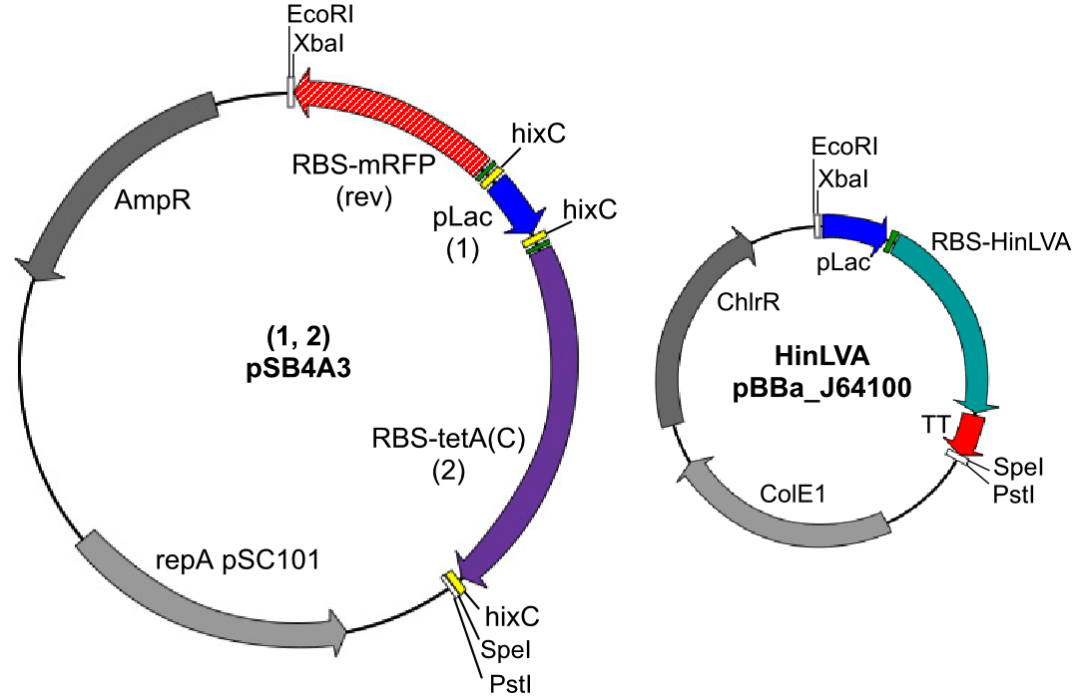
**BPP and HinLVA plasmid constructs**. A solved BPP plasmid (left) contains a flippable *pLac *promoter (blue arrow) and *RBS-tetA(C) *(green rectangle = *RBS*, purple arrow = *tetA(C)*). The *pLac *promoter is pancake 1 and *RBS-tetA(C) *is pancake 2. *hixC *sites (yellow rectangles) flank each flippable element. Inversion of *pLac *is detected by expression of the reverse (rev) upstream *RBS*-*mRFP *reporter. HinLVA is expressed from a second plasmid (right). AmpR = ampicillin resistance marker, ChlrR = chloramphenicol resistance marker, repA pSC101 and ColE1 = origins of replication, RBS = ribosome binding site, TT = double transcription terminator, white boxes = cloning sites.

To build a proof-of-concept model, we designed a two-pancake BPP containing the *Lac *promoter (*pLac*) and a tetracycline resistance coding region with a ribosomal binding site upstream (*RBS-tetA(C)*), each flanked by *hixC *sites (Fig. 2). Each configuration of this two-pancake stack is represented by a mathematical signed permutation. For instance, *hixC-RBS-tetA(C)-hixC-pLac*_rev_-*hixC *is represented as the signed permutation "*(2, -1)*" where *RBS-tetA(C) *is 2 and *pLac *is 1. The positive value (*2*) represents the forward orientation of *RBS-tetA(C) *and the negative value (*-1*) represents the reverse orientation of *pLac*, denoted *pLac*_*rev *_(*pLac *reversed). The eight possible signed permutations can be plotted as vertices of a graph (Fig. 1b). Two signed permutations are connected by an edge if it is possible to convert one permutation to the other with a flip of one or two pancakes. When flipping occurs at random, the starting permutation can be converted into any of its three neighboring permutations. In cells, after a given amount of time (*i.e.*, number of flips), flipping is stopped by manual cell lysis, BPP plasmids are purified and transformed into new cells lacking HinLVA, and solved BPP plasmids are detected by resistance to tetracycline (*pLac *driven *RBS-tetA(C) *expression) in each colony. The time point at which the BPP is first solved at random reflects the minimal number of flips required to solve the BPP.

### HinLVA flips and sorts *hixC*-flanked DNA segments *in vivo*

**Figure 3:**
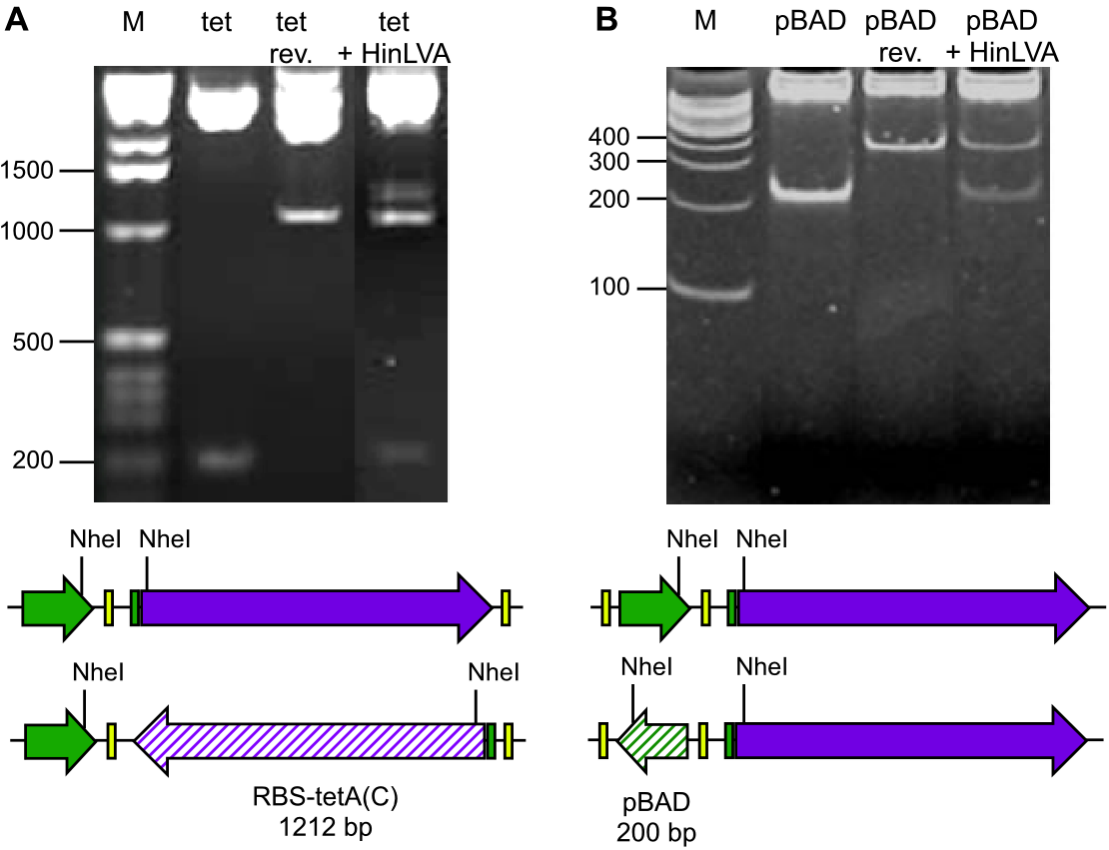
**HinLVA mediates inversions of short and long DNA fragments**. HinLVA-mediated flipping was assessed by NheI restriction digests. Gel electrophoresis images are shown in A and B. (A) A construct carrying *hixC*-flanked *RBS-tetA(C) *in the forward orientation (solid purple arrow) is characterized by a ~200 bp NheI restriction fragment (gel lane 2). The reverse orientation of *RBS-tetA(C) *(hatched purple arrow) yields a larger ~1100 bp band (lane 3). When forward oriented *hixC*-flanked *RBS-tetA(C) *is exposed to HinLVA, bands for both forward and reverse orientations of *RBS-tetA(C) *are detected (lane 4). (B) Similarly, forward oriented *hixC*-flanked *pBAD *promoter (solid green arrow) is converted to the reverse orientation (hatched green arrow) after exposure to HinLVA (lane 4). These data show that HinLVA-mediated inversion activity is not constrained by the length of *hixC*-flanked DNA.

In order to solve the BPP, HinLVA must be able to flip single pancakes of varying sizes, flip adjacent segments independently, and sort segments by flipping multiple pancakes simultaneously. First, we tested HinLVA-mediated inversion on single *hixC*-flanked DNA segments of different lengths. HinLVA successfully flips the 1212 bp *RBS-tetA(C) *segment (Fig. 3a). The length of *RBS-tetA(C) *is comparable to the segment that is inverted by Hin recombinase in Salmonella [9,10]. HinLVA can also flip the much shorter 200 bp *hixC*-flanked promoter (Fig. 3b). Restriction digest fragments indicate approximately equal molar amounts of both conformations (forward and reverse), suggesting that flipping of one DNA pancake has reached equilibrium ~24 hours after transformation. These data indicate that HinLVA-mediated inversion reconstituted in *E. coli *is not limited by fragment size, at least not within the range of 200 – 1212 bp.

**Figure 4:**
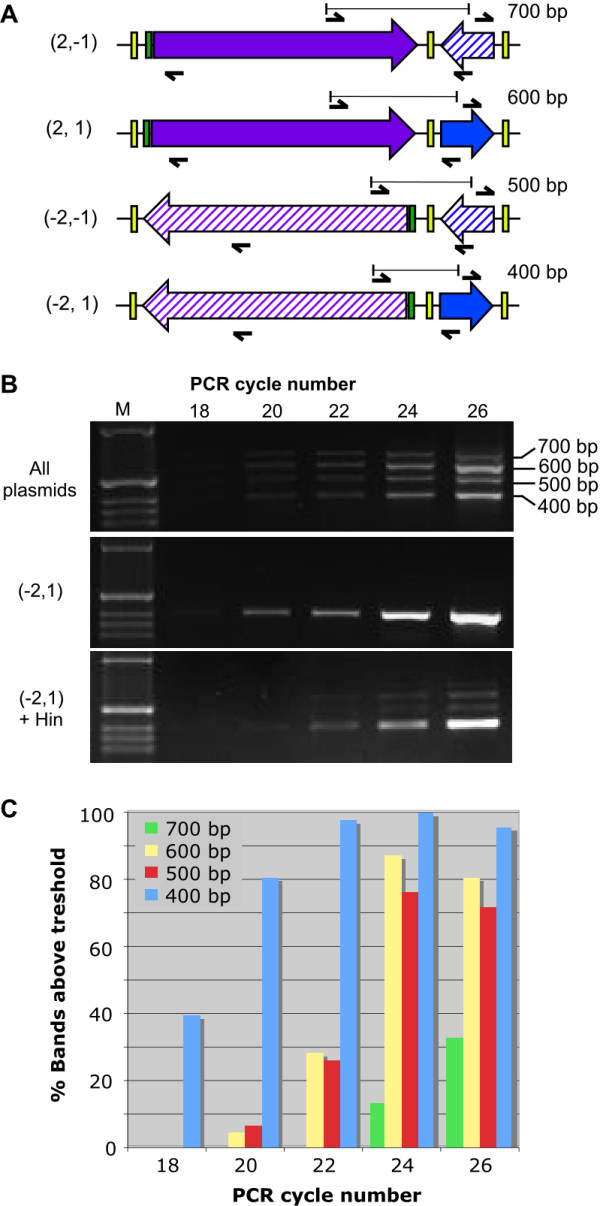
**HinLVA flips adjacent *hixC*-flanked segments independently**. (A) Four pancake stack arrangements can be distinguished by quantitative multiplex PCR. Black arrows indicate primers for multiplex PCR. Bars indicate sizes of PCR amplicons. Whole stack inversions that generate the other four possible arrangements cannot be distinguished in this assay. (B) Quantitative PCR of an equimolar mix of all eight manually assembled BPP plasmids (top row), BPP plasmid *(-2, 1) *alone (middle row), or a single 10-hour colony of cells carrying BPP plasmid *(-2, 1) *in the presence of HinLVA (bottom row). The first lane in each gel contains a DNA molecular weight marker (M). (C) Histogram showing the frequency of detectable bands at 18, 20, 22, 24 and 26 PCR cycles (n = 46 for each band at each cycle). At 10 – 11 hours following cotransformation of *(-2, 1) *and *HinLVA*, the starting pancake arrangement predominates (400 bp band). A single flip of either pancake alone (producing a 500 bp or a 600 bp amplicon) is next most frequent occurrence, while two successive flips (700 bp) is least common.

Next, we cotransformed cells with a BPP plasmid containing a *hixC*-flanked *RBS-tetA(C)*_*rev *_coding region and a *hixC*-flanked *pLac *promoter (permutation *(-2, 1)*) and a HinLVA expression plasmid (Fig. 2). The *RE *was omitted from the BPP plasmid to slow the rate of inversion. We used multiplex semiquantitative PCR (sqPCR) to monitor flipping of the two adjacent *hixC*-flanked DNA segments. Each of the four internal rearrangements can be detected by a sqPCR amplicon of a distinct size (Fig. 4a). Eleven hours after transformation, single colonies were picked for whole cell sqPCR. Bands from all four configurations were visible in samples where *(-2, 1) *was cotransformed with HinLVA (Fig. 4b). Flipping occurred in the absence of the *RE*, demonstrating that HinLVA and a pair of *hixC *sites are sufficient for a functional Hin/*hix *DNA inversion system in *E. coli*. The starting pancake arrangement *(-2, 1) *is the predominant plasmid in all colonies tested. Plasmids generated from a single flip of either *RBS-tetA(C) *(pancake 2) or *pLac *(pancake 1) are the next most frequent, while plasmids generated from two sequential flips of both pancakes 2 and 1 are the least common. We could not detect significant bias for flipping of the larger *RBS-tetA(C) *segment or the smaller *pLac *promoter (Fig. 4c), suggesting that flipping is not influenced by the size of the DNA segment.

The sqPCR results suggest that flipping has not yet reached equilibrium after 11 hours of HinLVA activity in the absence of *RE*. Plasmid supercoiling might be a limiting factor. Hin-mediated inversion requires a negatively supercoiled plasmid DNA substrate [15,16]. The loss of four negative supercoils after each inversion event [17] might require cells to undergo cell division to reset optimal supercoiling before a second inversion event can occur. Based on the 4 hour lag time and 36 minute maximum doubling rate of the cotransformed cells, we estimate that no more than 12 doublings occurred before sqPCR analysis. Twelve cell divisions appear to be insufficient to allow the distribution of rearrangements to reach equilibrium.

**Figure 5:**
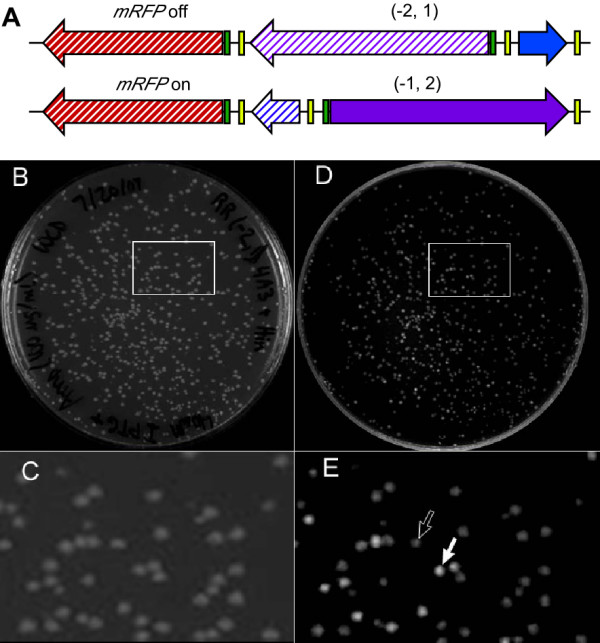
**HinLVA flips adjacent *hixC*-flanked segments simultaneously**. (A) A map of the BPP plasmid including the *RBS-mRFP*_*rev *_reporter followed by *hixC*-*tetA(C)*_*rev*_-*hixC*-*pLac*-*hixC (-2, 1) *is shown at the top. In this arrangement, *pLac *cannot drive *mRFP *expression. Simultaneous inversion of both segments converts *(-2, 1) *into *(-1, 2)*; the *pLac *promoter is directed towards *mRFP *so that *mRFP *expression is turned on. (B, inset C) White light photograph of colonies ~18-hours after cotransformation with BPP plasmid *(-2, 1) *and HinLVA. (D, inset E) mRFP protein production visualized under ultraviolet light (solid white arrow) indicates a simultaneous flip of two adjacent pancakes that placed *pLac *in the reverse orientation adjacent to *RBS*-*mRFP*_*rev*_. Some colonies do not glow red (open arrow), indicating a lack of double segment flipping or subsequent conversion into other arrangements that lack *mRFP *expression (*e.g.*, *(1, 2)*).

Finally, we assessed simultaneous inversion of both DNA pancakes. In order to accomplish this operation, Hin must recognize the outer-most *hixC *sites and ignore the central *hixC *site between the segments. Inversion of the entire permutation *(-2, 1) *generates permutation *(-1, 2) *in which the *pLac *promoter is repositioned to drive *mRFP *reporter expression (Fig. 5a). Inversion of the promoter alone, producing *(-2, -1)*, is insufficient to induce detectable levels of mRFP (Table 1). Colonies containing HinLVA and the *(-2, 1) *BPP plasmid were grown as a liquid culture then the Hin-exposed BPP plasmids were isolated and transformed into bacteria. About one third of the cell colonies appeared red (Fig. 5b) indicating that simultaneous inversion of both DNA segments occurred at a high frequency. Thus, HinLVA is capable of mediating the inversion of at least two adjacent flippable DNA segments.

**Table 1:** Two-pancake permutations and their phenotypes

BPP Plasmid construct^*a*^	Perm.^*b*^	mRFP expression on amp, IPTG^*c*^		Growth on tet, amp, IPTG^*d*^	
		expected	observed	expected	observed

*RR-hixC-pLac-hixC-RBS*-*tetA(C)*-*hixC*	(1, 2)	-	-	+	weak
*RR*-*hixC*-*tetA(C)*_*rev*_*-RBS*_*rev*_-*hixC*-*pLac*_*rev*_-*hixC*	(-2, -1)	+	-	+	+
*RR*-*hixC*-*pLac*-*hixC*-*tetA(C)*_*rev*_*-RBS*_*rev*_-*hixC*	(1, -2)	-	-	-	weak
*RR*-*hixC*-*RBS-tetA(C)*-*hixC*-*pLac*_*rev*_-*hixC*	(2, -1)	+	-	-	-
*RR*-*hixC*-*pLac*_*rev*_-*hixC*-*RBS-tetA(C)-hixC*	(-1, 2)	+	+	-	+
*RR-hixC-tetA(C)*_*rev*_*-RBS*_*rev*_*-hixC-pLac-hixC*	(-2, 1)	-	-	-	+
*RR-hixC-pLac*_*rev*_*-hixC-tetA(C)*_*rev*_*-RBS*_*rev*_*-hixC*	(-1, -2)	+	+	-	-
*RR-hixC-RBS-tetA(C)-hixC-pLac-hixC*	(2, 1)	-	-	-	+

*a *Two-pancake BPP constructs in plasmid pSB4A3.

*b *Signed permutation corresponding to each BPP construct.

*c *Cells carrying a BPP plasmid were selected on ampicillin (amp) plates. IPTG-induced expression of *mRFP *was visually detected as fluorescent red color (+).

*d *Serial dilutions of colonies showed normal (+), weak, or no (-) growth on tetracycline (tet) upon IPTG-induced *tetA(C) *expression.

### Modeling and detection of phenotypic output

**Figure 6:**
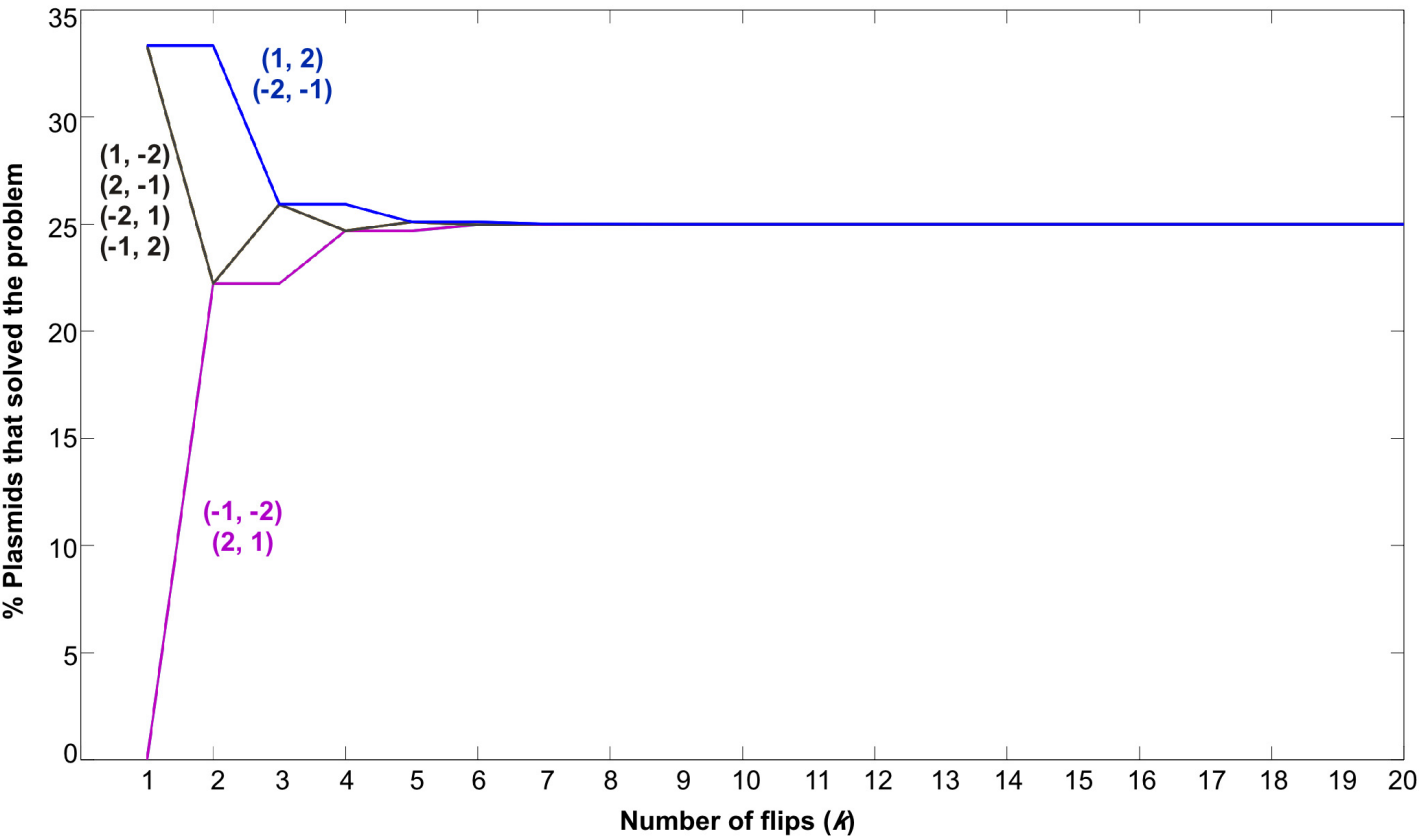
**Mathematical model of random flipping**. Flipping was modeled as a Markov Chain in which each of the possible eight starting permutations is a state (shown in parenthesis in the graph). At each step (Number of flips (*k*)) in a random walk on the graph in Figure 1b, the probability of a plasmid being properly sorted (% Plasmids that solved the problem) after *k *flips is calculated as the number of paths of length *k *from the initial state to the solution state, divided by the total number of paths of length *k *from the initial state to any state. Starting permutations that show equivalent behavior can be grouped into three families (distinguished by color in the graph). The families are distinguishable for up to 4 flips; at 5 flips and beyond they reach a state of equilibrium at 25% plasmids solved.

We sought to use the power and sensitivity of antibiotic resistance phenotype screening to detect solved BPP plasmids. sqPCR analyzes one colony at a time and requires several plasmids to generate a detectable PCR amplicon, whereas screening can rapidly distinguish a single solved BPP plasmid from millions of unsolved plasmids in a cell culture. Permutations *(1, 2) *and *(-2, -1) *both encode a functional tetracycline resistance gene that should allow cells to live in the presence of tetracycline. The other six permutations encode a disrupted tetracycline resistance gene and should lead to cell death in the presence of tetracycline. Based on these predicted phenotypic outputs, we designed a mathematical model of random flipping over time (successive flips) to predict how cell survival (the percentage of solved pancake stacks) might change over time. We modeled flipping as a Markov Chain in which each of the possible eight signed permutations is a state. Our model is synonymous with a random walk on the graph in Figure 1b. We assumed that any segment of DNA flanked by two *hixC *sites (pancake 1, pancake 2, or both) is equally likely to be flipped by HinLVA and that all flips in the population of cells happen synchronously. According to this model, the probability of a plasmid being properly sorted after *k *flips is determined by the number of paths of length *k *from the initial state to the solution state, divided by the total number of paths of length *k *from the initial state to any state. For instance, there are six paths of length 3 from initial state *(2, 1) *to solution state *(1, 2) *(Fig. 1b); because there are 27 possible paths of length 3 that start at *(2, 1)*, the probability of being in a solution state after three flips is 6/27 (22%). We observed two interesting features of the output from a simulation of random flipping (Fig. 6). First, the conversion of unsolved BPP plasmids towards and away from the solution state reaches equilibrium at 25% survival after five flips. Second, several starting arrangements show equivalent behavior as they approach equilibrium (*i.e.*, *(1, -2) *and *(-1, 2)*). The simulation output has implications for further design of our system. If our model is correct, only one representative from each class of equivalent starting configurations needs to be tested. Furthermore, if equilibrium (25% survival) is reached after only five flips, slowing Hin-mediated inversion by omitting the *RE *may be required to detect significant changes in cell survival over time.

As an initial step towards carrying out flipping *in vivo*, we manually constructed all eight pancake permutations (excluding the *RE*) and transformed them into cells to confirm their phenotypes. We observed several unexpected outcomes. In cells that contain a strong *pLac *repressor (lacI^Q^), BPP plasmids *(1, 2) *and *(-2, -1) *showed significant tetracycline resistance without activation of *pLac *by IPTG. We also observed that HinLVA-mediated inversion does not require induction of the *pLac *promoter on the HinLVA plasmid, indicating general leakiness of *pLac *promoter activity probably due to more lacI^Q ^binding sites than available repressor protein [18]. The addition of IPTG appears to slow the growth of *(1, 2) *transformants; this might be result of toxic TetA(C) over expression [19]. We expected to detect *mRFP *expression from all four plasmids that contain reversed *pLac*. However, reversed *pLac *fails to induce *mRFP *expression when it is positioned after *tetA(C) *(*i.e.*, *RBS-mRFP-hixC-RBS-tetA(C)-hixC-pLac*_*rev*_*-hixC*). Increased distance from *mRFP *or the DNA structure of *tetA(C) *[20,21] might block transcription of *mRFP*.

We found it surprising that four constructs in which *pLac *is not in the proper position and/or orientation to drive expression of *tetA(C) *were able to confer tetracycline resistance; in the presence of IPTG, three of these showed more robust growth than cells carrying *(1, 2)*. When the *pLac *promoter was removed from the construct, cells were still tetracycline resistant (data not shown), thus *pLac *is not required for expression in the pBR322-derived cloning vector we had been using (pSB4A3). Read-through transcription by RNA polymerase binding to the antibiotic resistance marker promoter or degenerate promoter sequences within the vector backbone [22] could result in *tetA(C) *expression in the tetracycline resistant scrambled permutations *(1, -2)*, *(-1, 2)*, *(-2, 1)*, and *(2, 1)*. We constructed an "insulated" vector (pSB1A7) containing forward and reverse double transcription terminator sequences to shield *RBS-tetA(C) *from read-through transcription. In pSB1A7, there was no expression of *RBS-tetA(C) *(forward or reverse) when *pLac *was removed from the construct. Arrangements *(1, 2) *and *(-2, -1) *produced tetracycline resistance, as expected. Surprisingly, we also observed tetracycline resistance in the insulated vector when *pLac *was reversed relative to *tetA(C) *in arrangements *(-1, 2) *and *(-2, 1)*, suggesting reverse promoter activity from *pLac*. Unlike forward transcription initiated from *pLac*, backwards transcription did not respond to IPTG as determined by cell growth; IPTG induction of forward transcription from *pLac *led to overexpression of *tetA(C) *and subsequent cell death [19]. Due to the backwards promoter activity of *pLac*, our manually built set of permutations are not distinguishable by phenotype, thus phenotype alone is insufficient to perform computation using *pLac *and *RBS-tetA(C)*. The observations described above demonstrate that the construction of synthetic biological devices can reveal unexpected characteristics of well-studied DNA elements (*e.g.*, *pLac*).

## Conclusion

We have demonstrated that a modified Hin/*hix *DNA recombination system can be used *in vivo *to manipulate at least two adjacent *hixC*-flanked DNA segments; HinLVA and *hixC *are sufficient for DNA inversion activity. The *RE *is not required, although it may play some role in preventing aberrant flips that lead to plasmid knotting [23] and subsequent plasmid loss [24]. Thus, the *RE *might be added to BPP plasmids to increase DNA recombination efficiency. Once phenotypic output is optimized for this system, the kinetics of flipping (*i.e.*, number of flips per unit of time) could be determined by comparing Markov Chain model simulation output to *in vivo *pancake sorting. Comparing actual cell survival to the survival probabilities predicted by our model should also enable us to determine whether flipping is biased for different sized DNA fragments.

The Hin/*hix *DNA recombination system could be used for other biological engineering applications. We have developed a set of modular genetic elements (*hixC*, *RE*, and *HinLVA*) that expands the repertoire of molecular tools for enzyme-mediated DNA manipulation *in vivo*. As with Cre/*loxP *from P1 bacteriophage [25] and Flp/*FRT *from yeast [26], Hin/*hix *may open avenues for recombinase-mediated transgene engineering. For instance, *hixC*-flanked promoters and other regulatory elements could function as flippable genetic toggle switches to regulate gene expression just as Hin mediates the expression of *flagellin *genes in Salmonella. Manipulation of genetic elements within a transgene at a single insertion site eliminates the problem of genomic position effects associated with independently introducing variants of transgenes at different loci. Furthermore, adjacent genetic elements could be rearranged at a single locus (*e.g.*, switching the positions of a promoter and transcriptional insulator to test how well the insulator blocks transcription). The ability of Hin recombinase to invert large and small DNA fragments and adjacent flippable elements demonstrates the potential flexibility of the Hin/*hix *system.

The capability of HinLVA to flip adjacent DNA segments indicates that this system could be scaled up to accommodate more complex pancake stacks. As an application in comparative genomics, flippable DNA segment arrays could serve as a model to improve our understanding of syntenic genome rearrangements that have occurred during evolution. Chromosomal regions exist as syntenic modules arranged in different orders and orientations in the genomes of related species. Each syntenic module can be considered a burnt pancake that has a particular order and orientation. Phylogenetic relationships between species can be inferred by using BPP mathematical modeling to compute the minimum number of rearrangements that link two syntenic genomes (see [27] for review). Hin-mediated rearrangements of an array containing different sized DNA fragments would help refine the mathematical model by accounting for the impact of differences in sequence composition and lengths of syntenic modules. Using Hin/*hix *to sort DNA fragment permutations *in vivo *expands the horizons for the emerging field of applied DNA-based computing.

## Methods

### Construction of parts and plasmids

All genetic elements reported here are standardized parts (prefixed "BBa_" and "pSB") that are flanked by universal "BioBrick" cloning sites [28] and are documented and distributed by the MIT Registry of Standard Biological Parts [29].*E. coli *strain JM109 was used for cloning and as the chassis for our system. The *recombinational enhancer *(*RE*, BBa_J3101) [30], *hixC *(BBa_J44000) [13], *RBS*_*rev *_(BBa_J44001) and *pBAD*_*rev *_(BBa_J44002) were assembled from 20 – 60 bp DNA oligomers that were designed using the "Oligo Cuts Optimization Program" [31]. *EcoRI *(5') and *PstI *(3') single stranded extensions were manually added to the terminal oligomers. An equimolar mix of single-stranded oligomers in 1× annealing buffer [100 mM NaCl; 10 mM Tris-HCl, pH 7.4] was incubated at 100°C for 5 minutes, then slowly cooled to ambient temperature in a water bath to produce double stranded DNA with *EcoRI *(5') and *PstI *(3') single stranded overhangs. The annealed DNA was ligated into linearized (*EcoRI *and *PstI *digested) cloning vector pSB1A2 using T4 ligase (Promega). *Hin *(BBa_J31000), *HinLVA *(BBa_J31001), and *tetA(C) *(BBa_J31007), and were cloned by polymerase chain reaction (PCR). Standard BioBrick prefix cloning sites (*EcoRI*, *NotI *and *XbaI*) or suffix cloning sites (*SpeI*, *NotI *and *PstI*) were included in the forward or reverse PCR primer, respectively. PCR amplicons were digested with EcoRI and PstI, electrophoresed in an agarose gel, purified, and ligated into a linearized cloning vector. *Hin *[GenBank: see Availability and requirements section for URL] was cloned from *Salmonella typhimurium *Ames strain TA100 genomic DNA using forward primer 5'-GCATGAATTCGCGGCCGCTCTAGATGGCTACTATTGGGTATATTC and reverse primer 5'-ATGCCTGCAGGCGGCCGCAACTAGTTAATTCATTCGTTTTTTTATAC. *HinLVA *was generated by PCR amplification of *Hin *(BBa_J31000) using forward primer 5'-TCTGGAATTCGCGGCCGCATCTAGAGATG and reverse primer 5'-CTGCAGGCGGCCGCTACTAGTATTAAGCTACTAAAGCGTAGTTTTCGTCGTTTGCAGCATTCATTCGTTTTTTTATAC containing a *ssrA *LVA degradation tag (based on *gfp*(down, LVA) [12]).*tetA(C) *was cloned from vector pSB1AT3 using forward primer 5'-GCATTCTAGATGAAATCTAACAATGCGCTCATC and reverse primer 5'-ATGCACTAGTTAGGTCGAGGTGGCCCGGC; the amplicon was cloned using a XbaI/SpeI digest. Reversed parts were generated by PCR amplification of MIT Registry parts using a forward primer containing *SpeI *and a reverse primer containing *XbaI*: *tetA(C)*_*rev *_(BBa_J31006) – forward 5'-ATGCACTAGTATGAAATCTAACAATGCGCTCATC and reverse 5'-GCATTCTAGATTAGGTCGAGGTGGCCCGGC; *pLac*_*rev *_(BBa_J31013) – forward 5'-ATGCACTAGTACAATACGCAAACCGCCTCTC and reverse 5'-GCATTCTAGAGTGTGTGAAATTGTTATCCGC; *mRFP*_*rev *_(BBa_J31008) – forward 5'-ATGCACTAGTATGGCTTCCTCCGAAGACGT and reverse 5'-GCATTCTAGATTAAGCACCGGTGGAGTGAC. Correct sizes and sequences of the genetic elements described above were confirmed by XbaI/SpeI double digestion and DNA sequencing. The constructs shown in Figure 2, the eight two-pancake BPP plasmids (Table 1), and the HinLVA expression vector were assembled using the standard BioBrick assembly method [27]. Proper assembly of construction intermediates was confirmed by EcoRI/PstI double digestion. Fully assembled two-pancake BPP constructs *(1, 2) *(BBa_S03684), *(-2, -1) *(BBa_S03681), *(1, -2) *(BBa_S03685), *(-2, 1) *(BBa_S03679), *(-1, 2) *(BBa_S03687), *(-2, 1) *(BBa_S03680), *(2, 1) *(BBa_S03677), and *(-1, -2) *(BBa_S03688) were confirmed by sequencing. BPP constructs were inserted into low copy pSC101 vector pSB4A3 (Fig. 2) and subsequently tested in insulated high copy pMB1 vector pSB1A7. *pLac-RBS-HinLVA-TT *(BBa_S03536) was inserted into low copy ColE1 vector BBa_J64100 (from Jeffrey J. Tabor) to create the HinLVA plasmid (Fig. 2). The cloning vectors are described in [29].

### Hin-mediated DNA recombination

Growth of transformed colonies on LB agar and in shaking liquid cultures took place overnight at 37°C. For single DNA segment flipping assays (Fig. 3), constructs containing the *HinLVA *gene on the same vector as the invertible segment (BBa_J44006 – *pLac*-*RBS-HinLVA*-*TT*-*hixC*-*pBAD*-*hixC*-*RBS-tetA(C)*-*TT*-*RE *or *pLac*-*RBS-HinLVA*-*TT*-*pBAD*-*hixC*-*RBS-tetA(C)*-*hixC*-*TT*-*RE *in vector pSB1A2) were transformed into JM109 chemically competent cells. After growth on selective media (LB agar plus 100 μg/mL ampicillin), single colonies were grown in liquid selective media (LB plus 100 μg/mL ampicillin) overnight. For the two-pancake stack flipping assay, 10 ng each of a plasmid containing a two-pancake stack (Table 1) and the HinLVA expression plasmid were combined in 5 μL sterile distilled H_2_O and co-transformed into Z-competent JM109 chemically competent cells according to the manufacturer's protocol (Zymo Research). Cells were grown at 37°C on LB agar selective media (20 μg/mL ampicillin, 50 μg/mL chlroamphenicol).

### Detection of DNA inversions

Inversions of single *hixC*-flanked DNA segments were detected by restriction digests (Fig. 3). Plasmid DNA was purified from each culture (Promega Wizard and Zymo Zyppy miniprep systems), digested with NheI, and resolved by agarose (Fig. 3a) or polyacrylamide (Fig. 3b) gel electrophoresis. Inversions of adjacent *hixC*-flanked segments were detected by multiplex semiquantitative PCR (sqPCR) (Fig. 4). 11 hours following transformation, 46 visible co-transformant colonies were picked from LB agar selective media and subjected to whole cell multiplex sqPCR (95°C, 10 min.; 95°C, 30 sec., 60°C, 30 sec., and 72°C, 15 sec. for 26 cycles). Each colony was suspended in 60 μL 1× PCR mix (Promega Green master mix plus 0.7 μM primers *pLacR *5'-GAATCGGCCAACGCGCGGGG, *pLacF *5'-GTTTCCCGACTGGAAAGCGG, *TetR *5'-GTAGAGGATCCACAGGACGG and *TetF *5'-TCGTAGGACAGGTGCCGGCA). 0.1 pmol of an equimolar mix of all 8 two-pancake BPP plasmids or 0.1 pmol of the *(-2, 1) *BPP plasmid alone was used as the template in a control PCR reaction. During PCR, an 11 μL aliquot was collected from each reaction after cycles 18, 20, 22, 24 and 26. Samples were electrophoresed on a 1.5% agarose gel and photographed under ultraviolet light using a BioRad imager. Band intensities were quantified using BioRad Quantity One imaging software. The detectable band threshold was set at 10,000 (after background subtraction).

To detect simultaneous flipping of two DNA segments (described in Fig. 5) single co-transformed colonies were picked from agar plates and grown in selective liquid media (20 μg/mL ampicillin, 50 μg/mL chlroamphenicol). To eliminate the Hin plasmid after flipping, plasmid DNA was purified from the liquid cultures and transformed into new competent cells. Cells were grown on LB agar containing 20 μg/mL ampicillin to select for transformants that contained a recombined BPP plasmid, and 40 μg/mL IPTG to induce *pLac*-driven expression of *mRFP*. *mRFP *expression was detected under ultraviolet light and photographed using a BioRad imager.

## Availability and requirements

Genbank: 

## Competing interests

The authors declare that they have no competing interests.

## Authors' contributions

LJH, TTE, JLP and AMC conceived the original study. KAH, TTE, and AMC constructed pancake plasmids containing *pLac*, assayed Hin-mediated two-pancake DNA inversions and drafted the manuscript. TTE and KAH assayed Hin-mediated single pancake inversions. LJH, JLP, WLH, JOD, and MB designed and executed mathematical modeling of the BPP and DNA flipping. KAH, BJO, SR, ADB, SS, TLB, EZ, AMC, TTE, ELJ, and KJM constructed and confirmed parts described in Methods (Construction of Parts and Plasmids). All authors read and approved the final manuscript.

## References

[B1] Adleman LM (1994). Molecular computation of solutions to combinatorial problems. Science.

[B2] Benenson Y, Paz-Elizur T, Adar R, Keinan E, Livneh Z, Shapiro E (2001). Programmable and autonomous computing machine made of biomolecules. Nature.

[B3] Soreni M, Yogev S, Kossoy E, Shoham Y, Keinan E (2005). Parallel biomolecular computation on surfaces with advanced finite automata. J Am Chem Soc.

[B4] Kossoy E, Lavid N, Soreni-Harari M, Shoham Y, Keinan E (2007). A programmable biomolecular computing machine with bacterial phenotype output. Chembiochem.

[B5] Gates WH, Papadimitriou CH (1979). Bounds for sorting by prefix reversal. Discrete Math.

[B6] Bafna V, Pevzner P (1995). Sorting by reversals: Genome rearrangements in plant organelles and evolutionary history of X chromosome. Molecular Biology and Evolution.

[B7] Hannenhalli S, Pevzner PA (1995). Transforming Cabbage into Turnip: polynomial algorithm for sorting signed permutations by reversals. Journal of the ACM.

[B8] Bourque G, Pevzner PA, Tesler G (2004). Reconstructing the genomic architecture of ancestral mammals: lessons from human, mouse, and rat genomes. Genome Res.

[B9] Zieg J, Silverman M, Hilmen M, Simon M (1977). Recombinational switch for gene expression. Science.

[B10] Zieg J, Simon M (1980). Analysis of the nucleotide sequence of an invertible controlling element. Proc Natl Acad Sci USA.

[B11] Johnson RC, Bruist MF (1989). Intermediates in Hin-mediated DNA inversion: a role for Fis and the recombinational enhancer in the strand exchange reaction. EMBO J.

[B12] Andersen JB, Sternberg C, Poulsen LK, Bjorn SP, Givskov M, Molin S (1998). New unstable variants of green fluorescent protein for studies of transient gene expression in bacteria. Appl Environ Microbiol.

[B13] Lim HM, Hughes KT, Simon MI (1992). The effects of symmetrical recombination site hixC on Hin recombinase function. J Biol Chem.

[B14] Moskowitz IP, Heichman KA, Johnson RC (1991). Alignment of recombination sites in Hin-mediated site-specific DNA recombination. Genes Dev.

[B15] Johnson RC, Bruist MB, Glaccum MB, Simon MI (1984). In vitro analysis of Hin-mediated site-specific recombination. Cold Spring Harb Symp Quant Biol.

[B16] Lim HM, Simon MI (1992). The role of negative supercoiling in Hin-mediated site-specific recombination. J Biol Chem.

[B17] Merickel SK, Johnson RC (2004). Topological analysis of Hin-catalysed DNA recombination in vivo and in vitro. Mol Microbiol.

[B18] Glascock CB, Weickert MJ (1998). Using chromosomal lacIQ1 to control expression of genes on high-copy-number plasmids in Escherichia coli. Gene.

[B19] Eckert B, Beck CF (1989). Overproduction of transposon Tn10-encoded tetracycline resistance protein results in cell death and loss of membrane potential. J Bacteriol.

[B20] Pruss GJ, Drlica K (1986). Topoisomerase I mutants: the gene on pBR322 that encodes resistance to tetracycline affects plasmid DNA supercoiling. Proc Natl Acad Sci USA.

[B21] Shishido K, Ishii S, Komiyama N (1989). The presence of the region on pBR322 that encodes resistance to tetracycline is responsible for high levels of plasmid DNA knotting in Escherichia coli DNA topoisomerase I deletion mutant. Nucleic Acids Res.

[B22] Sassone-Corsi P, Corden J, Kedinger C, Chambon P (1981). Promotion of specific in vitro transcription by excised "TATA" box sequences inserted in a foreign nucleotide environment. Nucleic Acids Res.

[B23] Heichman KA, Moskowitz IP, Johnson RC (1991). Configuration of DNA strands and mechanism of strand exchange in the Hin invertasome as revealed by analysis of recombinant knots. Genes Dev.

[B24] Deibler RW, Mann JK, de Sumners WL, Zechiedrich L (2007). Hin-mediated DNA knotting and recombining promote replicon dysfunction and mutation. BMC Mol Biol.

[B25] Gu H, Marth JD, Orban PC, Mossmann H, Rajewsky K (1994). Deletion of a DNA polymerase beta gene segment in T cells using cell type-specific gene targeting. Science.

[B26] Broach JR, Hicks JB (1980). Replication and recombination functions associated with the yeast plasmid, 2 mu circle. Cell.

[B27] Hayes B (2007). Sorting Out the Genome. American Scientist.

[B28] Knight T (2007). Idempotent Vector Design for Standard Assembly of Biobricks.

[B29] MIT Registry of Standard Biological Parts. http://partsregistry.org.

[B30] Perkins-Balding D, Dias DP, Glasgow AC (1997). Location, degree, and direction of DNA bending associated with the Hin recombinational enhancer sequence and Fis-enhancer complex. J Bacteriol.

[B31] Oligo Cuts Optimization Program. http://gcat.davidson.edu/IGEM06/oligo.html.

[B32] Genome Consortium for Active Teaching. http://www.bio.davidson.edu/GCAT.

